# Supplementary material for: Predicting visual function by interpreting a neuronal wiring diagram
Source: Nature. 2024 Oct 2;634(8032):113–23. doi: 10.1038/s41586-024-07953-5 (PMC11446822; doi:10.1038/s41586-024-07953-5)
Supplement: Supplementary file 6 — CRF and ERF predictions for individual TmY4 and TmY9 cells. Analogous to Supplementary Data 3, but for TmY target types. Shown are the top four monosynaptic pathways, the strongest pathway passing through each of the top ten intermediary types (ranking from Extended Data Fig. 7), and the trisynaptic pathway Tm1–TmY–Dm3–TmY (see the section entitled Prediction of spatial normalization). [file 41586_2024_7953_MOESM6_ESM.zip › DataS4/TmY9qΓèÑ/720575940618531867.pdf]

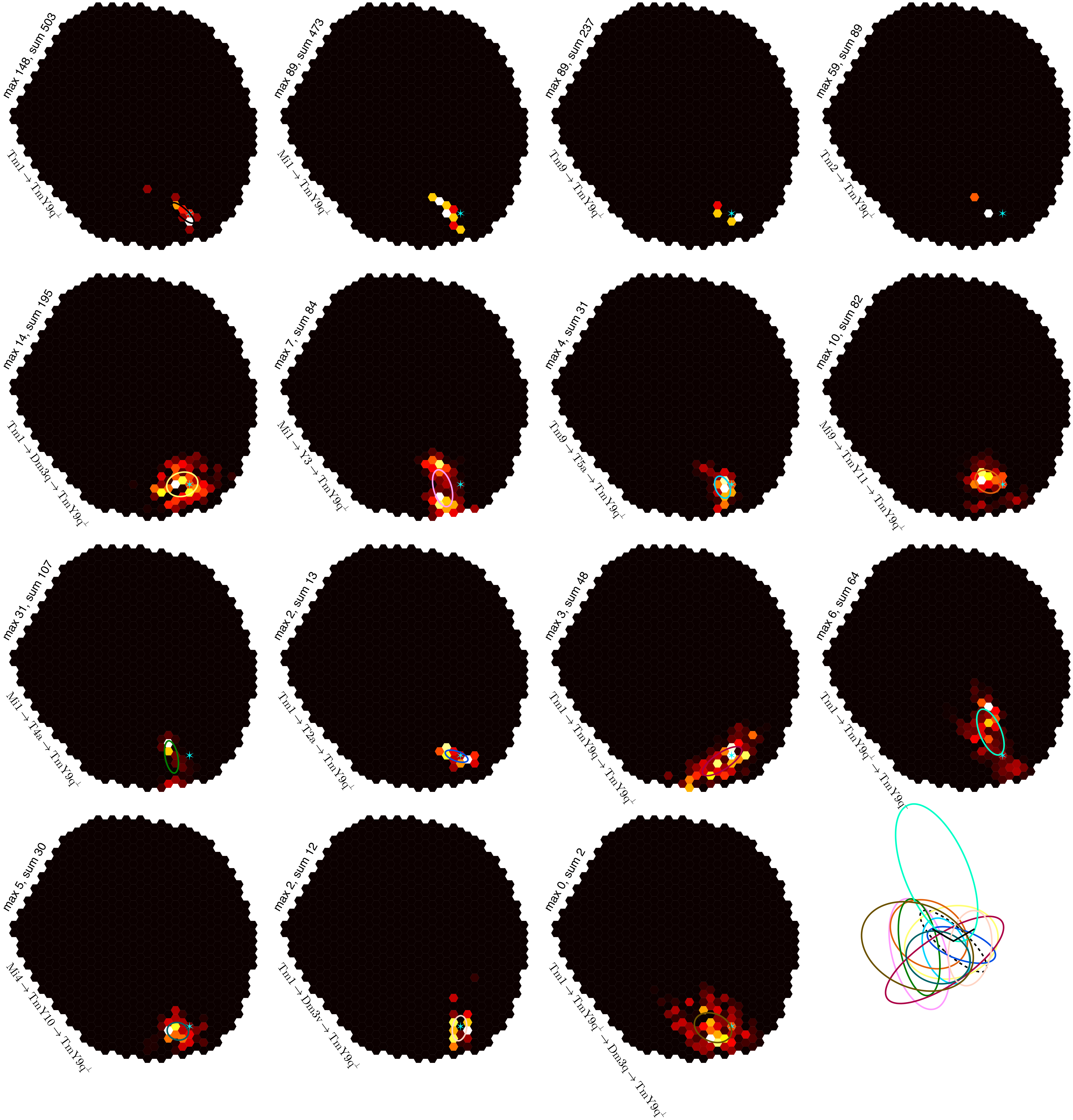
max 148, sum 503
Tm1 → TmY9q⊥
max 89, sum 473
Mi1 → TmY9q⊥
max 89, sum 237
Tm9 → TmY9q⊥
max 59, sum 89
Tm2 → TmY9q⊥
max 14, sum 195
Tm1 → Dm3q → TmY9q⊥
max 7, sum 84
Mi1 → Y3 → TmY9q⊥
max 4, sum 31
Tm9 → T5a → TmY9q⊥
max 10, sum 82
Mi9 → TmY11 → TmY9q⊥
max 31, sum 107
Mi1 → T4a → TmY9q⊥
max 2, sum 13
Tm1 → T2a → TmY9q⊥
max 3, sum 48
Tm1 → TmY9q → TmY9q⊥
max 6, sum 64
Tm1 → TmY9q⊥ → TmY9q⊥
max 5, sum 30
Mi4 → TmY10 → TmY9q⊥
max 2, sum 12
Tm1 → Dm3v → TmY9q⊥
max 0, sum 2
Tm1 → TmY9q⊥ → Dm3q → TmY9q⊥
